# Beyond adverse outcome pathways: making toxicity predictions from event networks, SAR models, data and knowledge

**DOI:** 10.1093/toxres/tfaa099

**Published:** 2021-01-22

**Authors:** Thomas Ball, Christopher G Barber, Alex Cayley, Martyn L Chilton, Robert Foster, Adrian Fowkes, Crina Heghes, Emma Hill, Natasha Hill, Steven Kane, Donna S Macmillan, Alun Myden, Daniel Newman, Artur Polit, Susanne A Stalford, Jonathan D Vessey

**Affiliations:** Lhasa Limited, Granary Wharf House, 2 Canal Wharf, Leeds, LS11 5PS, UK

**Keywords:** AOP, Reasoning, Predictive toxicology

## Abstract

Adverse outcome pathways have shown themselves to be useful ways of understanding and expressing knowledge about sequences of events that lead to adverse outcomes (AOs) such as toxicity. In this paper we use the building blocks of adverse outcome pathways—namely key events (KEs) and key event relationships—to construct networks which can be used to make predictions of the likelihood of AOs. The networks of KEs are augmented by data from and knowledge about assays as well as by structure activity relationship predictions linking chemical classes to the observation of KEs. These inputs are combined within a reasoning framework to produce an information-rich display of the relevant knowledge and data and predictions of AOs both in the abstract case and for individual chemicals. Illustrative examples are given for skin sensitization, reprotoxicity and non-genotoxic carcinogenicity.

## Introduction

### Introduction to AOPs

Adverse outcome pathways (AOPs) [1] were first developed in the 2000s to express the sequence of events leading from a molecule interacting with a biological target [known as a molecular initiating event (MIE)] to an adverse outcome (AO). The pathways include measurable key events (KEs) linking the MIE to the AO in a biologically plausible sequence and describe the evidence of the relationships between them as key event relationships (KERs). Importantly, the events were devised in the context of the level of organization at which the effect could be seen to occur, ranging from molecular through cellular and organ-level events to events affecting individuals and populations. The pathways were initially developed in the sphere of ecotoxicology [1] but subsequently the concept has been taken up more widely [2] and has been used for many toxicity endpoints—perhaps most successfully for skin sensitization [3]. The Organization for Economic Co-operation and Development (OECD) have taken a supportive role in reviewing and publishing diverse AOPs, now included in an online library [4], with work plans published for many others [5]; additionally, they have published guidelines in how to develop AOPs including questions to be asked when assessing the evidence for KEs and KERs [6]. Efforts have been underway for several years to standardize AOPs and their constituent parts (KEs and KERs) most notable of which is AOP-Wiki and the related projects in the Adverse Outcome Pathway Knowledge Base (AOP-KB) [7–9]. Standardization is important for several reasons: consistency of terminology is needed to reduce duplication; review of suggested AOPs allows for them to be put into the context of other, previously submitted or accepted AOPs. The development of a confidence framework allows the increasing use of AOPs for regulatory purposes [10].

### Limitations of AOPs

From the first attempts to define and codify AOPs there have been issues that are difficult to resolve. These include the fact that AOPs, by their nature, are agnostic of chemicals; that is to say that an AOP is devised such that no knowledge of the chemical that induces the first event (the MIE) is held in the AOP. This makes sense from a theoretical point of view but all experiments that are devised to observe the AOP in practice need a particular chemical to initiate the sequence; in practice, pathways are developed with prototypical compounds (stressors) known to give rise to the postulated sequence of events. In contrast, the complementary concept of mode of action (MOA) [11, 12] relates the activity of a particular chemical to the sequence of events that follow from its interaction with biological entities. There is much overlap in the concepts shared by AOPs and MOAs though the latter, being compound specific, includes consideration of a chemical’s metabolism and dose–response thus allowing them to be more relevant to toxicological risk assessment of the chemical under study.

AOPs are generally linear, simplified constructs; although they may be branched, they start from a single MIE and lead to a single AO. This is a somewhat artificial limitation as, in practice, it is known that a KE can give rise to several others which in turn lead to a variety of adverse events, i.e. KEs are shared between pathways. It is therefore potentially helpful to consider the knowledge about a KE in the context of many or all pathways leading from a KE; this gives rise to key event networks (KENs).

### The need for going beyond AOPs

The framework and concepts of AOPs have demonstrated their utility as shown, e.g. by the contributions from many individuals and organizations to AOP-Wiki [8]. However, the concepts need augmentation if the knowledge they represent is to be utilized more fully in the prediction and understanding of the toxicity of individual compounds, compound classes or other subsets of chemical space. In this paper we consider how a scientist might make use of the combination of knowledge about AOPs, assays related to KEs and (quantitative) structure activity relationships ((Q)SAR) predictions to assess toxicological hazard. We show how software can be used to support a scientist in these assessments. We do not consider specific work flows of these scientists but typically toxicology assessments are made either in early discovery of chemicals and pharmaceuticals, their development or prioritization or for submission to regulators. In each case a different level of detail or evidence is required: in early stages many chemicals are considered and software would be required to support high-throughput screening whereas for regulatory submission detailed evidence and considerations are required. In the former case, software can be used to quickly screen out chemicals and/or rank those that remain; providing that the means of screening out or prioritising is trusted then it is unlikely that detailed information on the basis of the decision is scrutinised (though should be available). In the latter case software can be used to help expert review. In between are scientists with one or a few lead compounds who have to consider assay and related data and consider how a chemical structure might be improved to obviate potential toxicity. Clearly, in all scenarios the most frequent use case would be ‘What does my compound do?’ and there are many aspects to the answer to that question. We illustrate three use cases that contribute to `What does my compound do?’ where AOPs can be used to provide a reasoned answer: the first might be asked as part of making a regulatory submission; it, along with the second and third use cases, may also be asked by discovery scientists considering a potential lead candidate.

### Use case 1: what do compounds like mine do?

In the context of AOPs this equates to what AOs do compounds like mine cause? The use of AOPs allows the answer to such a question to be more convincing by demonstrating which MIEs and KEs are observed by compounds similar to the one of interest. The observations of KEs are, of course, by proxy through assay data where a measurement conducted as part of an assay can be linked to a KE (or MIE). The user can then frame the assay observations in the context of the AOP or wider network.

In the context of ‘compounds like mine’ the question of compound similarity arises. There are many different ways of determining chemical similarity, often a measure of similarity will be based on chemical features or descriptors which can be compared via a Tanimoto coefficient. However, more pertinent similarity measures may be available—such as a biological similarity measure—that may better represent activity relevant to an MIE or KE. In fact, this is what makes AOPs, and associated networks, an attractive approach for the problem. Different similarity measures can be used which are appropriate to the KE in question rather than using a single generic method. Fragment-based focussed similarity may be more useful for reactive KEs whereas pharmacophoric type descriptors would be more appropriate for KE involving protein active site binding.

### Use case 2: given this assay result, what AOs should I be worried about?

This question is answered by relating assay information in general—rather than about specific compounds—to KEs and by way of the AOP or KEN to one or more AOs. The `assay result’ mentioned in the use case may be an individual measurement taken as part of an assay or an `overall call’ derived from several measurements taken as part of a bioassay protocol. The measurement may be one that other assays share and may be indicative of different AOs: for instance a measurement of an increase in organ weight may be found as part of a rodent carcinogenicity assay. If the organ were the uterus the observation of weight gain may be indicative of a developmental and reproductive toxicology (DART) AO rather than hyperplasia which might lead to carcinogenicity.

As an assay measurement may be associated with a KE some distance from the MIE of an AOP, it is not necessary (though it might be useful) to know about all the preceding events in the pathway. Knowledge of preceding events is particularly important when considering human relevance of a pathway where the meaning of an assay result may be questioned in the context of the pathway. Also, as the KE(s) that an assay can be linked to may be in different AOPs, the answer to this question may well cover several AOPs.

### Use case 3: if I have this assay result, what assay should I run next?

Here the question is about confirmatory assays and/or assays which are most discriminating in coming to a conclusion about the likelihood of observing an AO. Underlying the question are considerations about how likely is it that, for a result giving rise to concern, it will lead to the AO. Furthermore, knowing which pathway is likely to have led to this KE might give the user enough information to dismiss the AO in the human context. For practical purposes, `what assay should I run next?’ will be tempered by cost and convenience of the assay and by availability of the compound in question; the question might be rephrased as `what assay should I run next (other than a long-term animal study)?’

Answering this question requires an understanding of which KEs are expected to follow from the one linked to the assay result that the user has, and how previously tested compounds have behaved. The question can be answered without specific knowledge of the structure of the compound of interest—though if this is known then the answer to the question can be augmented with similarity measures that are used to answer Question 1.

**Figure 1:**
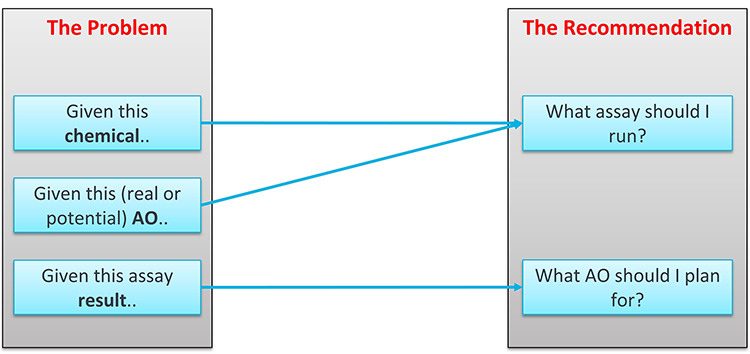
Use cases under consideration in this paper.

The use cases are summarised in Fig. 1.

### Approaches to reasoning: combining expert knowledge and data

Decision making about toxicological hazard and risk involves combining a lot of different sorts of evidence, including (Q)SAR, *in vivo*, established *in vitro*, new and emerging *in vitro* (biomarkers and omics data). Experts may spend a lot of time using their existing knowledge to combine this evidence and reach a conclusion to generate new knowledge.

It is difficult to reason in a consistent and reproducible way without structure to the evidence: we are ‘drowning in information, while starving for wisdom’ [13]. This is where AOPs provide a framework to structure reasoning between different pieces of evidence.

Many (Q)SARs and other models for predicting toxicity give their predictions and the user makes reasoned decisions about how predictions from QSARs and other models are taken into account: ICH M7 is a good example of this encoded in regulatory guidance [14]. Adding predictions and data to an AO network allows us to put many different data and predictions together and look specifically at diverse data and predictions for specific KEs or AOs. Furthermore, we can use the strength of KERs between an event of interest, its upstream and subsequent events to relay a considered likelihood through the network. Therefore, we can then consider diverse and independent inputs about the likelihood of a KE occurring and reason between them. We can use expert knowledge about the reliability and applicability of assays, observations and predictions related to a KE to come to an overall conclusion of the likelihood of the KE occurring.

When considering assay data, we might take into account the reproducibility of the assay, the known variation in the measurements or the source of a particular measurement and how well we believe the assay reports for a KE occurring. Furthermore, we might want to consider known limitations of an assay—what might be termed an assay applicability domain or, negatively, ‘gotchas’—where some combination of the compound under study or the assay conditions (cell line, solvent, dose etc.) mean that the assay result is unreliable. Additionally, we might want to consider the biological complexity of the assay system and use this to weight the emphasis put on the result as well as the relationship between the assay, the KE and the AO. When considering a prediction, we would take into account the performance of the prediction model, its applicability domain and all the usual provisos when considering a statistical or expert system prediction. In the case of both assays and predictions we would take into account, if known, the performance in the immediate chemical space around a particular chemical under study.

In this paper, we illustrate how we address these issues with illustrations from prototype software designed to address the above use cases. The prototype helps us discuss how software can help in resolving issues encountered in this space without discussing them in the context of a specific, commercial piece of software.

## Materials and Methods

### Data

In the prototype software, observations made as part of assays came from several sources depending on the endpoint. All the data are publicly available.

### Skin sensitization data

Data were taken from Urbisch *et al*. [15] with further elaboration from the authors and in-house curation. The curated dataset contains a total of 210 chemicals structures, with data from six different skin sensitization assays. Of the 210 chemicals, 197 have i*n chemico* data from the direct peptide reactivity assay (DPRA); 203 have *in vitro* data from the KeratinoSens™ and/or Lusens assays; and 175 have *in vitro* data from the h-CLAT and/or U-SENS™ assays. The U-SENS™ results were collected from a number of recent publications [16–18] as the prediction model for this assay has changed since the original data were published. All 210 chemicals have *in vivo* data from the local lymph node assay (LLNA), as well as an expert-derived human skin sensitization classification.

### Carcinogenicity data

Data were compiled in house from public data made available in Lhasa Limited’s Vitic software; additional data were used from a dataset from Kirkland *et al.* [19, 20] and a collection of *in vitro*/*in vivo* chromosome aberration and micronucleus test data derived from the FDA/CFSAN/OFAS knowledge base [21]. Compounds were selected that had data associated with one of 12 assays identified as being relevant to KEs or AOs associated with carcinogenicity. Unreliable results and those where there was not a clear result (Positive, Negative or Conflicted) were excluded. A summary call for the compound was taken if there was not a detailed experimental protocol available in Vitic.

Chemical structures were standardised using in house software which performs the following functions:

Curation—identifies and checks for common structural problems (e.g. representation of transition metal complexes).

Validation—checks for valency violations etc.

Normalization—attempts to fix common resonance problems and generate a single tautomer for a given compound class.

Biological data associated with the assays were curated for consistency, in terms of metabolism (e.g. Rat S9, S9 Rat, Rattus S9, etc.), result call (Positive, positive, active, etc.) and exposure time. This was required due to inconsistencies between the datasets.

Where a compound has been tested in the same assay more than once (using all the assay variables) an overall call was generated according to the scheme:

Multiple calls (Positive, Negative and/or Conflicted) => Conflicted.

Positive => Positive

Negative => Negative

Conflicted => Conflicted

A compound can still have more than one result if the assay definition was different (e.g. different exposure time or metabolising enzyme etc.).

### Developmental and reproductive toxicology data

Assay data came from the publically available data stored in the Vitic database.

During AOP creation, data from assays were identified during literature and database searches. The assays are reviewed and then mapped to relevant KEs in the AOP network.

The rules for curating the bioactivity data for assay vary depending on the type of assay and data availability. Data for assays were reviewed and classifications are given in accordance with the assay guidelines. Typically, this means looking for specific activity and accounting for any non-specific responses, e.g. cytotoxicity for *in vitro* assays, or systemic toxicity for *in vivo* assays.

For generating overall calls, expert-defined rules are established and then applied to summarize the assay measurements. For example, such an approach was designed to generate a set of classifications for *in vitro* measurements for chemical interactions with the oestrogen receptor [22].

### Knowledge

Expert knowledge about KEs, KERs, assay reliability, the connections between assays and KEs, the importance of different measurements undertaken as part of the assay, links between predictions and KEs, and expert judgements on the reliability and human relevance of all of these were added to the prototype software. For the covalent protein binding leading to skin sensitization AOP these elements were taken from AOP Wiki. All other AOPs and associated knowledge were compiled in-house by the authors and details of these will be given in forthcoming publications, some of which have appeared in preliminary form [23–26]. Although the prototype tool allows for data entry and editing, bulk data and knowledge were imported into the prototype’s underlying relational database using SQL scripts which in turn were generated from KNIME [27] workflows developed in-house. The KNIME workflows consume datasets prepared from Vitic and public data sources as well as expert-prepared documents containing the AOP knowledge in Excel or simple text formats; the KNIME workflows marshal the data in SQL format.

### (Q)SAR predictions

Derek Nexus predictions described come from version 5.0.1 of Derek Nexus [28] in version 2.1 of the Lhasa Knowledge Suite. Predictions were extracted from a Setaria [29] repository which stored the Derek Nexus output and entered into the prototype software database using SQL scripts developed in KNIME. The prototype database was then augmented with links between Derek Nexus predictions—specifically the alerts fired or, in the case of skin sensitization, the prediction of no activity—and one or more AOs, KEs or assays; the links between alerts and AOs, KEs and assays were hand-curated by the authors using knowledge of how the alerts were constructed in the first place. Typically, when a Derek Nexus alert is derived it will be based on several different types of study, involving different assays and testing regimes. Alerts are, therefore, typically associated with an AO. But in some cases the alert has been derived from results from a narrower field and so we can relate it to a KE (which Derek Nexus then extrapolates to the AO using knowledge which may include the basis for the AOP). In some cases, alerts have been derived from extensive *in chemico* or *in vitro* assays which allows us to relate the alert to that assay in the software prototype.

### Software

The data and knowledge in the preceding sections were entered into a database linked to prototype software written with Lhasa’s Discovery suite [30]. The figures in this paper are taken as screenshots from this prototype, with additional labels to features mentioned in the text where necessary.

## Results

We report the integrated display of AOP knowledge with assay information and (Q)SAR predictions as successive augmentation of a basic design implemented in the software prototype. Some of the motifs in the display correspond to concepts expressed in AOP Wiki [8] and other display tools such as Cytoscape [31] where the user has a degree of control on how concepts and attributes are depicted. Clearly where similar concepts are represented the only value in using a different notation are for reasons of user ease and limitations associated with the choice of display. For instance, representing KEs as circles—as AOP Wiki does—makes it more awkward to put much text in them in an interactive display in computer software; our prototype uses tooltips for detail (captured as labels in the subsequent figures) but, in practice, this may be found to impede the ease of use of the software.

### Graphical display of AOPs and networks

**Figure 2:**
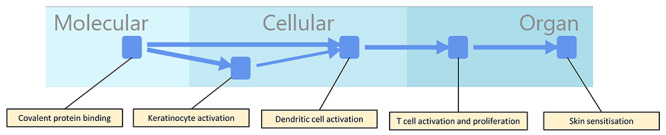
The AOP for covalent protein binding leading to skin sensitization. KEs, including the MIE and AO are represented as rounded squares, and KERs by the lines between them. KEs are labelled for reference with the text.

Figure 2 shows the display of the network for the AOP for skin sensitization initiated by covalent protein binding [32]. Certain labels are added to the network for identification purposes relevant to this discussion; in the context of dynamic usage of a software solution the labels would appear as pop-ups. One of the issues in presenting AOPs and the information about them in a graphical format is the trade-off between showing all the information and the usability of the display. An alternative way of identifying the network components is to label them all, though this necessitates more zooming and panning by the user to find areas of interest.

Covalent protein binding leading to skin sensitization is a well-developed and accepted AOP [3, 32] which is relatively straightforward in having only three intermediate KEs between the MIE and the AO. Figure 2 shows the MIE, AO and intermediate KEs as rounded squares and the KERs as directed lines (arrows) between them. Note that the organizational level of the KEs is indicated using swim lanes so that the initial covalent protein binding MIE is shown in the molecular level swim lane whereas the skin sensitization AO is shown in the organ level swim lane and intermediate KEs are in the cellular level and organ level swim lanes. Although the left-to-right ordering of the KEs indicates their causal relationships, the size of the distance between the KEs (and subsequent embellishments) is arbitrary and optimised for display only; similarly, arrangement on the vertical axis is arbitrary. The software prototype uses an implementation of the Sugayama framework [33] in order to layout the AOPs and KENs; the implementation is deterministic—it will produce the same layout every time—though the addition or deletion of nodes (representing KEs) from the network can alter the layout extensively.

The thickness of the arrows representing the KERs is used to convey the weight of evidence of the KERs and it can be seen that the arrow representing the KER between keratinocyte activation and dendritic cell activation—for which the evidence is considered weaker than the other KERs [13]—is shown as being narrower than other KERs in Fig. 2. Using arrow thickness to convey the weight of evidence has some drawbacks: there will only be a limited number of thicknesses which can be readily distinguished by the user and, even then, they will only be clear if there are a variety of thicknesses in the diagram for comparison, or if there is an easily accessible legend by which the user can interpret the arrow thickness and thus the weight of evidence it represents. It nevertheless is probably the clearest visual cue for representing the weight of evidence.

### From AOPs to networks

**Figure 3:**
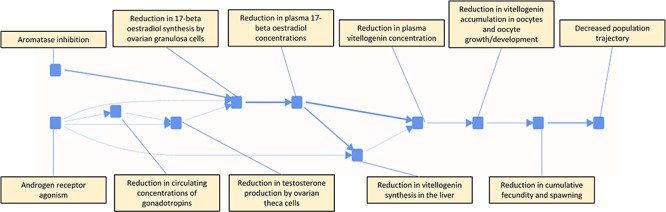
Network generated by combining several individual AOP’s for a common endpoint, reproductive toxicity.

Figure 3 shows our first step in elaborating the display of KEs from a linear straightforward AOP, as it shows a network of two AOPs towards a common AO: aromatase inhibition leading to reproductive toxicity and androgen receptor agonism leading to reproductive toxicity. These two pathways converge at the KE of reduction in 17-beta oestradiol synthesis by ovarian granulosa cells. The same notation is used in Fig. 3 for KEs and KERs as in Fig. 2, however, there are two MIEs rather than just one in the pure AOP view. Note that the information in Fig. 3 is now less simple than the AOP in Fig. 2 and the issues mentioned above concerning display of detail versus user scrolling and panning are more accentuated. Note also the network nature of the pathways—that KEs which are shared between individual AOPs become nodes of splitting and joining in the network. In terms of knowledge management it is important that KEs appearing in multiple pathways are named consistently. The visualization of AOP networks (AOPNs) has previously been made available through Cytoscape [31, 34, 35] using the AOPXplorer extension [9, 36].

### Augmentation with assays

**Figure 4:**
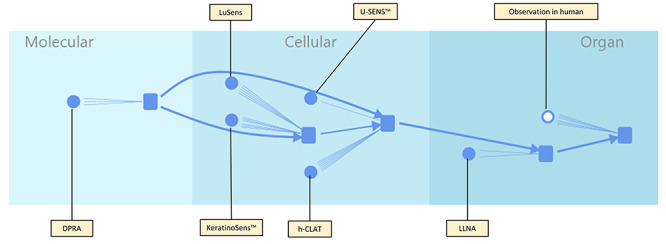
Addition of assay information to the basic AOP diagram.

Assays are represented in Fig. 4 by circles and are associated with KEs. Each line between an assay and a KE represents a particular type of measurement undertaken as part of the assay. For example, the DPRA [37] has three lines connecting it to the covalent protein binding MIE, one each for cysteine depletion, lysine depletion and overall call. The LLNA [38] has two lines connecting it to the T-cell proliferation and activation KE4, one each for EC3% value and overall call. The other assays; KeratinoSens™ [39], LuSens [39], h-CLAT [40, 41], U-SENS™ [41], LLNA and Observation in human have differing numbers of lines related to how many measurements are used in the assay. Other skin sensitization assays are available which could also be mapped to the KE in this AOP, however, as these were not present in our dataset these were not included in this example. Information about assays is further enriched by an expert-assigned reliability which is represented by the thickness of the circle border: a fully filled circle represents a highly reliable and reproducible assay. Circles with thinner walls (i.e. larger amounts of white in the middle) are less reproducible/reliable assays. In this diagram, the right-most assay which is connected to the skin sensitization AO is for human data and is (somewhat controversially!) considered less reproducible and therefore has a large white centre. In contrast the left-most assay is the OECD-validated *in chemico* DPRA and thus very reproducible indeed so is represented with a fully filled circle.

### Further augmentation with (Q)SAR predictions

**Figure 5:**
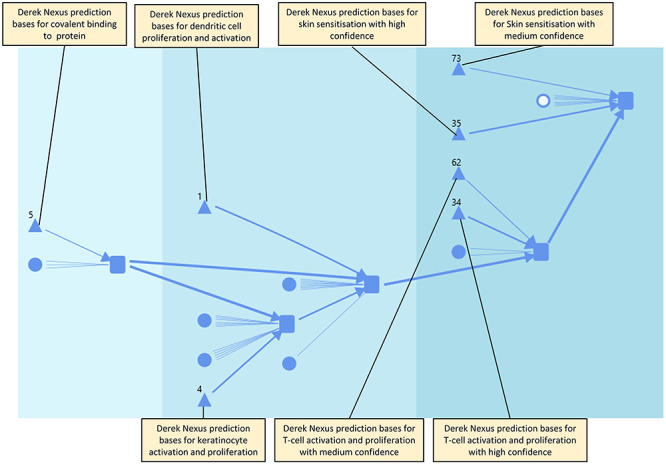
Further augmentation of the knowledge diagram by (Q)SAR predictions, shown as triangles.

(Q)SAR predictions are shown in Fig. 5 by triangles; predictions can be made for assay observations or for KE occurrence. In Fig. 5, the predictions are exclusively Derek Nexus predictions and the number next to each triangle shows how many bases for prediction there are for that KE/AO. Typically, each basis for prediction will be a Derek Nexus alert but this is augmented by other factors that Derek Nexus takes into account, for instance the species about which the prediction is made; furthermore, some Derek Nexus alerts predict for more than one event. The association of an alert (as a basis for prediction) with an assay indicates that the assay data have informed the writing of the alert. The predictions based solely on Derek Nexus alerts are asymmetrical in that the presence of an alert indicates a positive (concerning) prediction for an assay but the reverse is not true: the absence of an alert does not indicate a negative (non-concerning) prediction for an assay or KE. Furthermore, Derek Nexus can make negative predictions for the skin sensitization AO in the absence of an alert and this is augmented with a confidence depending on whether or not Derek Nexus is aware of features in a submitted molecule that it are novel or are known to be associated with molecules that it misclassifies. This means that although there are 100 alerts for skin sensitization in the Derek Nexus knowledge base (version 2020.1), the numbers on all the arrows add up to more than this. Different bases for prediction have different weights of evidence associated with them; in this case the differences are alerts which lead to a plausible outcome in Derek Nexus and those which lead to an equivocal outcome. The difference in the weight of evidence associated with the prediction is represented in the line thickness. For example, in the dataset used, there are 35 bases for prediction which lead to a plausible outcome for skin sensitization (the AO represented by the right-most square) and these are connected by a thicker line than the 73 bases for prediction which lead to an equivocal prediction for skin sensitization.

### Addressing the use cases

With assays and predictions connected to a network of KEs and AOs it is possible to address the use cases above.

Use Case 1: What do compounds like mine do?

As stated above, the fundamental issue with this question is defining compound similarity; this is a subjective concept and the issues involved in using different measures has been discussed several times [42–44]. We illustrate the issue in the prototype software where there are several measures of similarity that can be used: a structure fingerprint where similarity between compounds is measured with a Tanimoto coefficient—that itself can be based on several different structural fingerprints; substructure and a simple biological fingerprint where compounds are considered similar if they fire the same Derek Nexus alerts. The other half of the question is then which activities is the user interested in. In this case we can use the AOP concepts of MIE, KE and AO to direct the user’s search.

**Figure 6:**
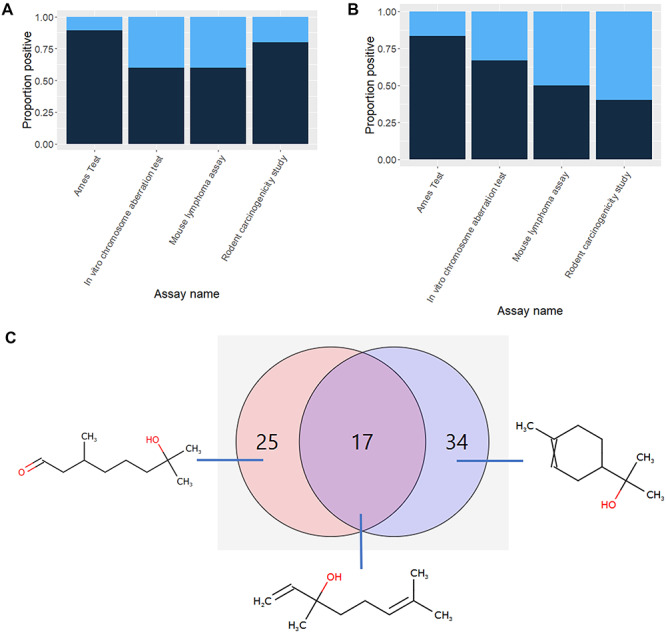
Activity of compounds considered similar to (+/−)-linalool in assays related to KEs in the carcinogenicity-related pathways. (**a**) Shows results for 42 compounds considered similar by a reduced graph pharmacophore fragmentation. (**b**) Shows results for 51 compounds considered similar by the Ceres fingerprint. In (a) and (b) dark blue represents overall positive assay results and pale blue represents overall negative results. In (**c**) the overlap between compounds in the two sets is shown: the 42 compounds shown in (a) are depicted in red and the 51 compounds shown in (b) are depicted in blue. The most similar compounds in the different areas are also shown.

Figure 6 shows a histogram of activity of the activity of compounds similar to (+/−)-linalool in four assays associated with KEs in carcinogenicity pathways with similarity measured using a Tanimoto coefficient based on two different fragmentation algorithms. The histogram has been normalized for the number of compounds so that the distribution of positive and negative assay calls can be more easily compared. In (a) a pharmacophore reduced graph fragmentation algorithm is used, whereas in (b) the in-house Ceres fingerprint algorithm [30, 45] is used. In (c) a Venn diagram shows that a total of 76 compounds have been selected with only 17 in both similarity sets. In (c) the most similar compound in each sector is also shown. Clearly the most similar compound that is in both sets is the compound linalool itself, but unique to the Ceres fingerprint similarity is the ring-containing structure—considered less similar by the pharmacophore-based similarity measure. In contrast, the aldehyde is unique to the pharmacophore similarity set. In general, the Ceres fingerprint does not discount similarity due to the presence of rings whereas the pharmacophore reduced graph favours skeletal similarity. It should be borne in mind, of course, that the chemical space occupied by compounds which have actually been tested will be innately ‘lumpy’ as tests may have been reported for a series of similar compounds. It is outside the scope of this discussion to consider how to normalize the occupied chemical space to correct for this distribution bias, but is another factor that users need to take into account when looking for trends in compounds from data which are considered similar. Notwithstanding these caveats, the difference in activity of compounds considered similar can be seen in the proportion of similar compounds testing positive in the assays, where the greatest discrepancy is seen in the proportion of compounds testing positive in the rodent carcinogenicity studies. Although neither measure of similarity should be considered more ‘correct’, or even more appropriate, the figure does illustrate how careful a user should be when using similarity measures.

### Use Case 2: given this assay result, what AOs should I be worried about?

**Figure 7:**
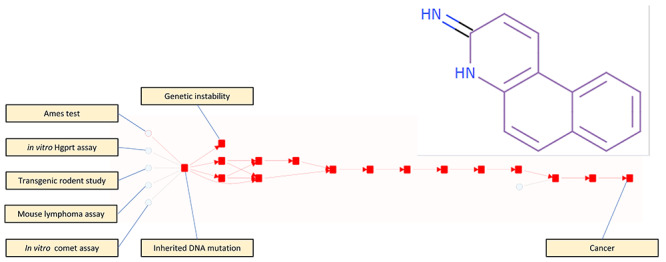
AO’s alerted from a positive Ames test result for the imine shown.

Figure 7 shows the conclusions that might be drawn from a positive Ames test result for the imine shown in the figure. The Ames test is directly linked to the inherited DNA mutation KE which leads to the genetic instability and cancer AOs.

In the figure, other assays related to inherited DNA mutation are also shown, but there are no results for the imine concerned.

In some ways the result is trivial in that there is only one assay result and no counter arguments so the positive Ames result cascades through all KEs to the AOs of genetic instability and cancer. Furthermore, the link between inherited DNA mutation (the KE for which Ames test reports) and both of the AOs is fairly well established, however in other cases the link between an assay and an AO may not be so apparent. In answering the question ‘which AO(s) should I be concerned about’ the scheme shown in Fig. 7 gives a qualitative answer, i.e. it gives information about hazard rather than risk and is an expression of the assay result and therefore is independent of the compound under study. By the same token, this makes the conclusion of hazard for the imine shown rather general and unsatisfactory and other knowledge, e.g. about the mechanism by which imines cause mutation, would be required in order that a user be satisfied that the prediction of hazard is made with a full understanding of the situation. We can enhance the hazard estimation by considering a statistical relationship between any particular assay result that we have and the occurrence of an AO—itself measured by an assay: this is considered in Use Case 3.

### Use Case 3: if I have this assay result, what assay should I do next?

**Figure 8:**
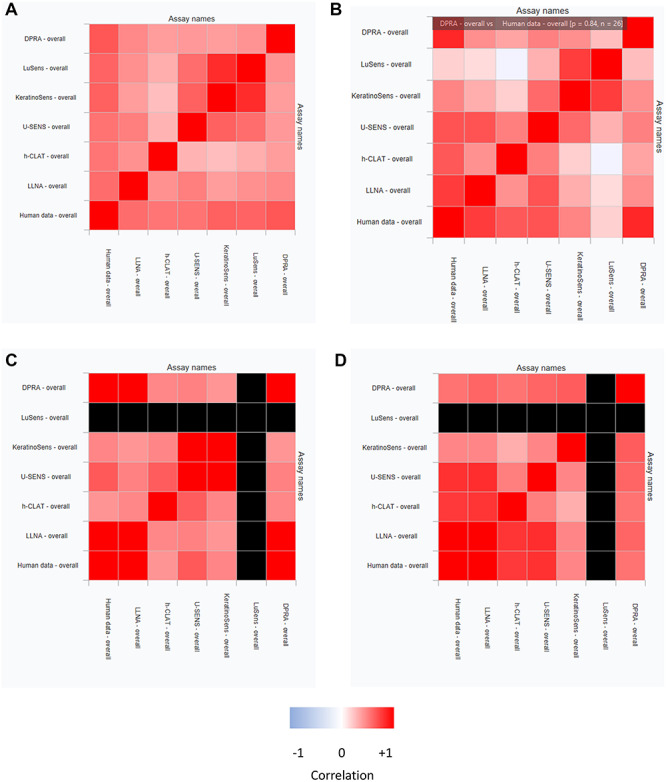
Simple heat maps showing the relationships between assay observations of different compounds. (**a**) The whole dataset (**b**) Compounds considered similar to the one under study (**c**) Compounds similar to the one under study which have a known positive response in the LuSens assay (**d**) compounds similar to the one under study which are negative in the LuSens assay.

Figure 8 shows a set of simple heat maps of the correlation of positive and negative calls in the assays associated with the skin sensitization pathway. The heat maps show strong positive correlation in red, strong negative correlation in blue; pale colours indicate little correlation and black indicates too few points to make a correlation. Figure 8(a) shows the general trends for all compounds in the data described above. Unsurprisingly, assays which are designed to report about the same KE show a strong correlation, with each other but there is good correlation between all the assays for the chemical space of the compounds in the database taken as a whole. Figure 8(b) takes a subset of the dataset in question where the compounds are considered to be similar to the putative compound under study—in this case 2, 4-dihydroxyaniline. The measure used for determining similarity in this case is a structural similarity based on a Tanimoto coefficient of a Ceres fingerprint. It allows the chemical context to be taken into account when considering assay response similarity. It can be seen the correlations are more varied. For example the correlation between DPRA and human results are very good for the compounds like the one under study: the software allows the user to hover over a square in the heat map to bring up the value of the correlation (0.84 in this case) and the number of compounds on which the correlation is based (*N* = 26 in this case). On this basis we may consider that a positive DPRA result is sufficient to conclude that the compound will be a skin sensitizer in humans.

**Figure 9:**
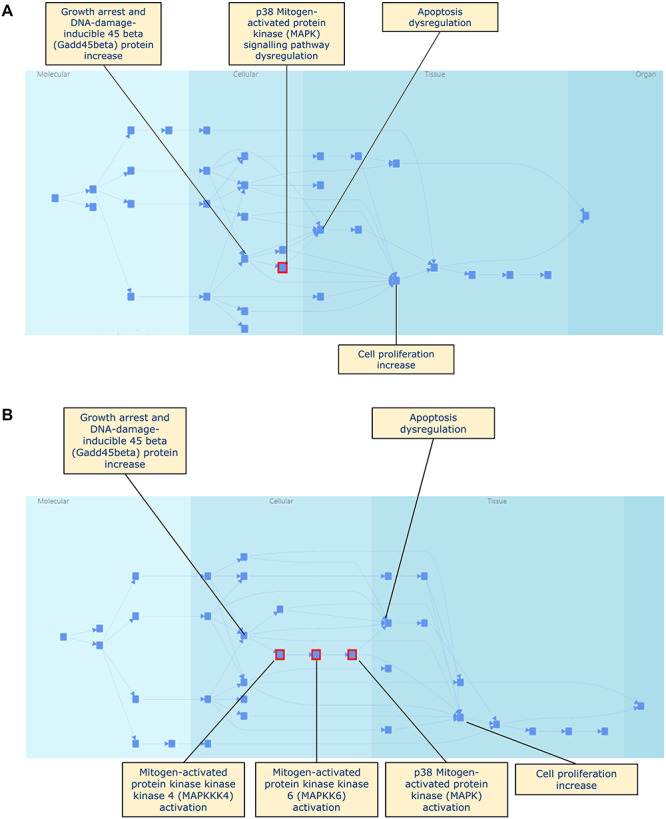

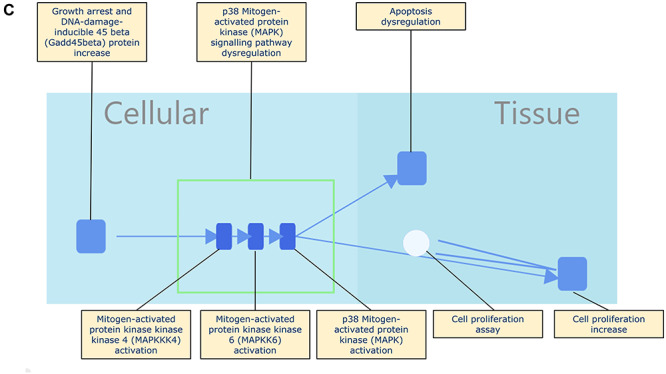
(**a**) The Retinoid X receptor binding leading to carcinogenicity AOP displayed with all KEGs collapsed; (**b**) the same AOP with expansion of the p38 MAPK signalling pathway dysregulation KE group; (**c**) a close up of the p38 MAPK signalling pathway dysregulation KEG and its sub-KEs.

However, we can note that the correlations of LuSens results for the compounds in this area of chemical space appear to be weaker than those for all compounds shown in (a). In Fig. 8(c), the data are pre-filtered so that only those compounds with a positive result in the LuSens assay are considered; note that this results in there being no correlations with LuSens in Fig. 8(c) and the associated column and row are black. Now we see strong correlations between DPRA, LLNA and human assays and also between KeratinoSens™ and U-SENS™ assays, with weaker correlations between assays outside these subgroups. In Fig. 8(d) only those compounds which are similar to the one understudy and with a negative LuSens result are shown. This reveals poor correlation between DPRA and KeratinoSens™ with the other assays, U-SENS™, h-CLAT, LLNA and human data, the four of which correlate quite well together. In summary, although the LuSens results as a whole correlate poorly for compounds similar to 2, 4-dihydroxyaniline, a particular LuSens result indicates which other assay might be untaken to get a good prediction of the human skin sensitization response.

### Further elaboration of KENs (i): Key event groups (KEGs) and representing pathway knowledge at different levels of detail

One of the challenging aspects of using both simple AOPs and KENs to represent knowledge and aid utilization of it for decision making purposes is capturing and expressing it to a user at an appropriate level of detail. In particular, an acceptable level of detail needs to be present to make a persuasive case that the knowledge of the KEs and their relationships is sufficient to allow regulatory decisions to be made. The level of detail associated with AIOPs or KENs is necessarily less than that of, say, cell signalling pathways but the underlying knowledge is connected and the appropriate level of detail to display or express will vary depending on the user’s needs. For instance, when describing a cell-based KE, it is possible, and sometimes desirable, to consider each of the sub-cellular KEs which together generate the event at the higher level of organization. In terms of capturing and reasoning about KEs, it is essential to focus on what is measurable. New and emerging methods and assays which measure things at a much more precise level, relating to protein expression e.g. commercially available biomarker assays, as well as omics data, allow for the subcellular processes to be investigated and the events that they report on captured within the context of a higher level KE.

This hierarchical relationship in the KEs can be captured and displayed and this is illustrated in Fig. 9, which shows that AOP of Retinoid X receptor binding leading to carcinogenicity. In Fig. 9(a) the whole network is shown; it contains several groups of KEs which are displayed in their collapsed (i.e. condensed) state. One of the KE groups, p38 Mitogen-activated protein kinase (MAPK) signalling pathway dysregulation, is selected and highlighted in red by the prototype software in the Figure; KEs leading to and from this KEG are labelled in the Figure. In Fig. 9(b), the p38 MAPK signalling pathway dysregulation KEG has been expanded and it can be seen that it contains three sub-KEs, also highlighted in red by the prototype software as belonging to the selected KE group. Note that the algorithm for laying out the network makes it difficult to compare the two networks by eye and the complexity of the network illustrates the points made above about the ease of getting an overview of the network whilst simultaneously having access to the detail. Software to support investigation and understanding of such complex networks must allow a user to focus on different areas in a dynamic way.

In Fig. 9(c) the software shows a close up of the KE group, its immediate neighbouring KEs and the sub KEs for easier investigation by the user. Note that through Fig. 9 one of the neighbouring KEs, cell proliferation increase, is itself an event group which could be expanded as well as the p38 MAPK signalling pathway dysregulation KE group.

### Further elaboration of KENs (ii): use of ontologies in relating KEs

**Figure 10:**
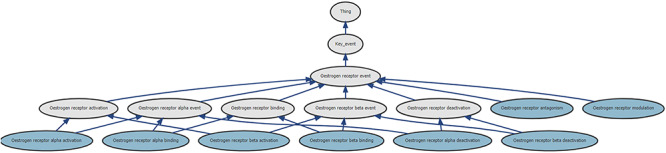
Ontobrowser display of an ontological relationship between KEs related to oestrogen receptors. Terms in dark blue represent KEs in the database, pale blue terms are not in the database and capture appropriate aggregations of more the more specific terms. The figure is taken from an SVG file generated by Ontobrowser with highlighting added separately.

As described above, KEs can be defined at different levels of detail and when combining KEs from different pathways this will cause some overlap between events. One way to capture the overlap and other relationships between KEs is to use an ontology. Using ontological annotations in organising KEs has been discussed by others [46] and in Fig. 10, we illustrate how KEs in the database of the software prototype relating to oestrogen receptors might be organised. The prototype software equates some of the terms in the ontology, which is stored in an Ontobrowser instance [47, 48], with KEs and thus allows the user to select sets of KEs which are related to a term in the ontology.

The ontological relationships in Fig. 10 as all ‘is a’ relationships and we can see that, for instance, the term ‘oestrogen receptor alpha binding’ has this relationship to be the ‘oestrogen receptor alpha event’ and the ‘oestrogen receptor binding’ terms. In the prototype software ontology terms are linked to KEs in the database so that the user can select groups of events which have a link to an ontological term; the selected events can then be viewed in the KENs using diagrams shown in previous figures.

Other groups have developed ontologies for use with AOPs that are more extensive. Burgoon [49] created the AOPOntology which is allied to a prediction system for AOs based on likelihood of KEs; an ontology related to developmental toxicity leading to spina bifida was developed [50, 51] and predictions for steatosis were made using Bayesian methods [52]. A different ontology for use with AOPs has been suggested by Ives *et al*. [53] in which KEs are described using event components of Process, Action and Object where each of these are populated by other ontologies or controlled vocabularies. In this context, we present Fig. 10 only as an illustration of how ontologies may be used for KEs and do not advocate development of further conflicting or competing ontologies.

We can also use an ontology approach to deal with assays. In particular, where assays are non-standard or where there may be many variations on in-house assays different by dosing regimen or observations that are nevertheless closely related and whose conclusions—rather than whose observations—might be useful to compare. It may also be convenient to classify standard assays according to an ontology. For example, a battery of tests from Toxcast [54] for oestrogen agonist activity suggested by Judson *et al*. [55] might be associated with terms in an assay ontology shown in Table 1. The entries in Table 1 form a very small bioassay ontology; EMBL-EBI provides a more extensive bioassay ontology which is under active development [53].

**Table 1:** Suggested ontology terms for a selection of assays for oestrogen agonist activity

Toxcast assay identifier	Ontology term
ACEA_T47D_80hr_Positive	Oestrogen receptor (ER) activation assay
ATG_ERE_CIS_up	Oestrogen receptor (ER) activation assay
Tox21_ERa_LUC_BG1_Agonist	Oestrogen receptor (ER) activation assay
Tox21_ERa_BLA_Agonist_ratio	Oestrogen receptor (ER) activation assay
OT_ERa_EREGFP_0480	Oestrogen receptor (ER) activation assay
OT_ERa_EREGFP_0120	Oestrogen receptor (ER) activation assay
ATG_ERa_TRANS_up	Oestrogen receptor (ER) activation assay
NVS_NR_bER	Oestrogen receptor (ER) binding assay
NVS_NR_hER	Oestrogen receptor (ER) binding assay
NVS_NR_mERa	Oestrogen receptor (ER) binding assay
Tox21_ERa_BLA_Antagonist_ratio	Oestrogen receptor (ER) deactivation assay
Tox21_ERa_LUC_BG1_Antagonist	Oestrogen receptor (ER) deactivation assay
OT_ER_ERbERb_1440	Oestrogen receptor (ER) dimerization assay
OT_ER_ERbERb_0480	Oestrogen receptor (ER) dimerization assay
OT_ER_ERaERb_1440	Oestrogen receptor (ER) dimerization assay
OT_ER_ERaERb_0480	Oestrogen receptor (ER) dimerization assay
OT_ER_ERaERa_1440	Oestrogen receptor (ER) dimerization assay
OT_ER_ERaERa_0480	Oestrogen receptor (ER) dimerization assay

The prototype software allows the use of ontologies to aid the user in focussing on relevant knowledge. As both knowledge and ontologies expand, this approach should become increasingly beneficial for making the knowledge tractable and expandable.

### Reasoning—incorporating assay observations and compound predictions

AOPs were originally devised to be agnostic of chemistry, and of chemical compounds in particular. The succession of events brought about by exposure to a particular compound is described in the related concepts of the modes or mechanism of action (MOA). Here, we seek to unite these two concepts by considering the sequence of KEs described in a network and moving from the chemically agnostic knowledge shown in Figs 2–5 to the specific knowledge concerning an individual chemical. In doing so we consider how to assess the evidence both about KEs and KERs and report a level of belief in them; reasoning about evidence, applying weight of evidence approaches and use of modified Bradford Hill criteria for assessing the evidence have been proposed previously in this context [10, 56]. In this paper, we consider categorical, rather than continuous, assignment of outcomes; inevitably this can lead to some hard cut-offs and edge cases which have a large impact on the conclusion. Although we acknowledge this disadvantage inherent in such a scheme, we think that the precision required to produce a continuous set of values associated with outcomes cannot be justified given the subjective nature of some of the arguments and evidence.

**Figure 11:**
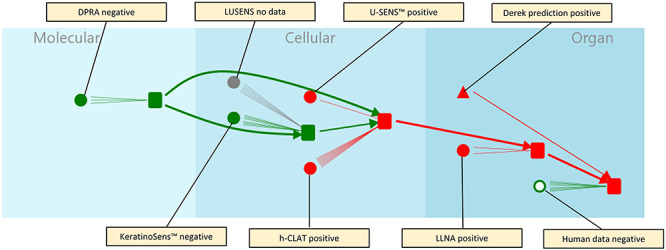
Showing assay results and structure activity relationship predictions for (+/−)-linalool and reasoned conclusions of likelihood through the pathway.

Figure 11 represents all the knowledge about the compound (+/−)-linalool in the underlying database associated with the software prototype. Importantly—and for the purpose of illustration—data which reported skin sensitization caused by an oxidised form of linalool is observed frequently in human patch tests and which caused a rewriting of the guidelines for linalool [57, 58] were not made available to the prototype.

Figure 11 can be compared with the abstract case shown in Fig. 5; potential predictions which have not been recorded are removed and colour has been added to show the assay observations and predictions. Red indicates a cause for concern—typically a positive response in an assay or a prediction of hazard—with green representing no cause for concern. The presence or absence of a cause for concern is evaluated at each node (assay, KE or prediction) and is transmitted through the network to the successive KEs via KERs. Where there are conflicting observations, a simple conservative call is made, i.e. where a KE has both cause for concern (red) and no cause for concern (green) inputs an overall call of cause for concern (red) is made; note that alternatives to this logic are discussed below. Figure 11 shows that for (+/−)-linalool, assay results relating to events early in the pathway are negative—coloured green to indicate no suggestion of hazard—whereas assays and predictions towards the end of the pathway are positive—coloured red to indicate a hazard. The lines between the assay and KE represent the different measurements that are made in the assay and are only coloured if they alone influence the colour of the downstream event; in practice only the ‘overall calls’ are coloured. Where no assay data are available, the assay is coloured grey and indicates to the user a possible confirmatory assay if one were required.

It is important to be aware of what the representation shown in Fig. 11 actually means. The colouring of the nodes and lines on the graph indicate the empirical evidence of the assay and prediction results. The assay results which do not indicate a cause for concern, e.g. the green DPRA result, do not imply that the mechanism of skin sensitization is other than through covalent protein binding—merely that the assay which aims to detect this event has suggested no cause for concern. This may be a limitation of the assay (a ‘gotcha’) or some other aspect of the mechanism of toxicity not captured in the knowledge. In the case of (+/−)-linalool, it is known that the skin sensitization comes from an oxidation product so the DPRA may not represent conditions comparable to an *in vivo* exposure. Indeed an advantage of the collation of assay results as presented in Fig. 11 is the indication of inconsistency in the data and this implies that there is more to the story than the assay results alone can tell.

In Fig. 11 all arguments are considered to contribute equally when resolving the cause for concern at any KE. Note that, as described above, the thickness of the lines representing the KERs is an indication of the essentiality of the KER in the AOP. The essentiality of the KE is one characteristic that we might like to consider when considering the cause for concern at a point in the network based on previously occurring KEs. Meek *et al.* [56] have described a detail scheme for assessing the weight of evidence relating to MOAs in terms of modified Bradford Hill criteria and give examples of how these can be used to reason about the believability of the pathway itself. These considerations can be brought together to form an innate level of confidence that a cause for concern at one KE in the pathway/network is passed on to the next. For the particular case of the AOP in Fig. 11, i.e. covalent protein binding leading to skin sensitization, the KEs and KERs are considered well established and there is no reason to reduce the contribution of the argument coming from a preceding KE in this AOP due to uncertainty in the truth of KER. Clearly this will not be the case in many AOPs, particularly those under development, therefore any reasoning scheme must be able to capture the weight of evidence at any KE and moderate the strength of argument that comes from it by taking into account the level of confidence in the KER. In practice, though, assuming there are no reasons for completely discounting an indication of hazard, the user will always want to be informed of it and may choose to use a conservative reasoning paradigm by default.

Although the reasoning in Fig. 11 shows a simple conservative call, as was outlined above, there are many facets to the reasoning that we might wish to consider—in particular the weight given to the argument; whether individual pieces of evidence are independent; whether the assay protocol has been run appropriately or is appropriate for the chemical class; the biological complexity of the test system and possibly how far down the pathway is the KE being measured. In other reasoning systems the direction and magnitude of arguments for and against a conclusion are considered. For example, we can devise a system in which a strong argument in favour of a conclusion can outweigh a weak argument against, and so on [38].

**Figure 12:**
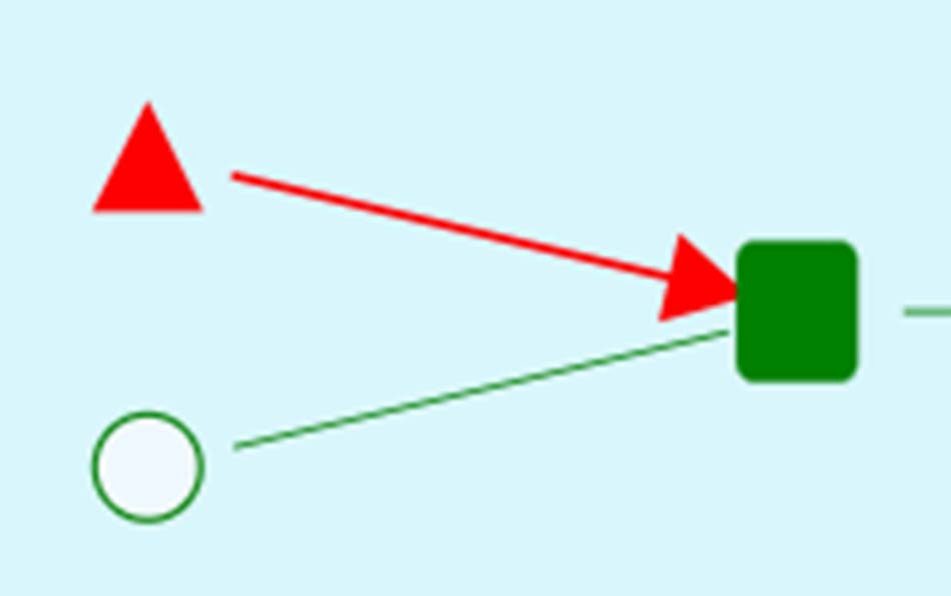
Alternative reasoning paradigm where assay results overrule predicted results. An assay result showing no cause for concern conflict with a prediction showing a cause for concern; the key event is not considered to be a cause for concern.

A feature of the reasoning as shown in Fig. 11 that may cause disquiet is the equanimity with which assay and predicted values are treated, but this is not the only logic that can be used. In Fig. 12, we illustrate an alternative approach in which assay results are always used in preference to predicted results: here an assay which is considered to have low confidence and illustrated in green to indicate the assay result gives no cause for concern overrules a prediction which does give rise to a cause for concern. This case replicates that in Fig. 11 for the reasoning about the skin sensitization AO. Furthermore we can weight assays by their biological complexity, with results from *in vivo* assays overruling those from *in vitro* assays and both overruling results from *in silico* predictions [25, 26].

**Figure 13:**
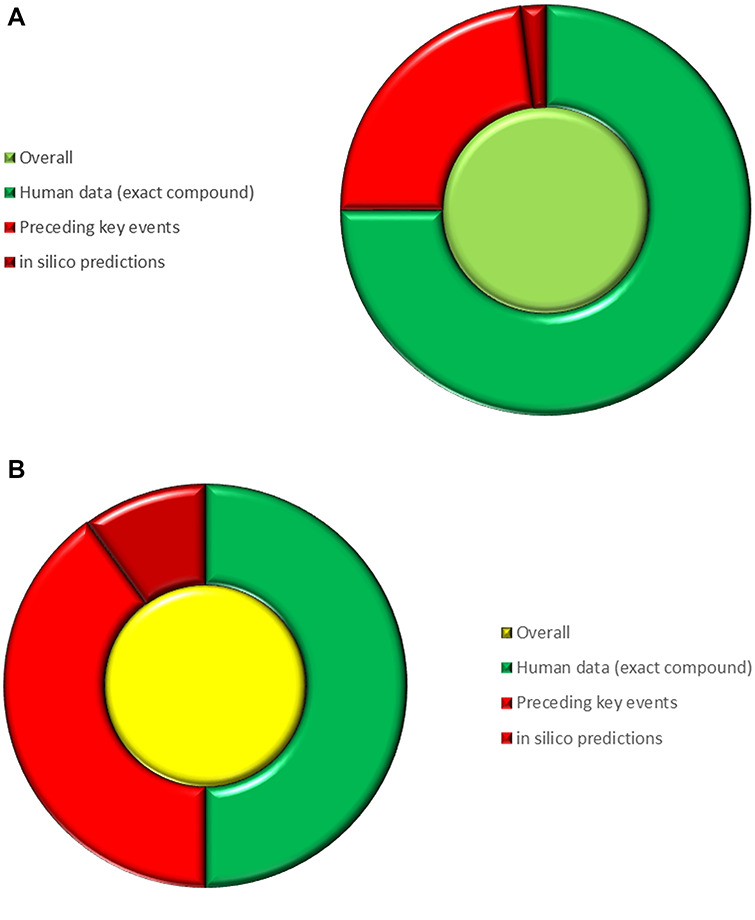
Illustrating different weightings of arguments for and against a conclusion of skin sensitization by (+/−)-linalool and showing an overall call.

In Fig. 13, we illustrate a simple, visual way of weighting arguments to come to a more nuanced conclusion. Different arguments are weighted and represented as slices of a pie chart, and an overall call is represented by the colour of the pie chart centre; the figure shows how to consider arguments leading directly to skin sensitization in humans by (+/−)-linalool shown in the right-most step in Fig. 11. The weighting of the individual components is made by a human expert and endeavours to reflect how much their own belief in the overall assessment would be influenced by the associated factors.

In Fig. 13, the three different lines of argument leading to the skin sensitization endpoint considered are: human data for the exact compound, likelihood of preceding KEs and *in silico* predictions, and the relative weighting of each argument is represented by its share of the outer ring. Although only three lines of argument are shown in this example, others might also exist; e.g. human data in related compounds, predictions of skin penetration etc. In Fig. 13(a), the human data are given the largest weighting over likelihood of preceding KEs and *in silico* predictions 75:23:2, respectively; in Fig. 13(b) the weightings are 50:40:10. The colour of each segment represents the level on concern that arises from the associated evidence on a scale from dark green indicating no concern to dark red indicating great concern with yellow indicating equivocation. The overall call is then represented by the colour of the centre circle: the variation in weighting between Fig. 13(a) and (b) gives a different overall call.

In practice, the user of the system might weight the arguments on a case-by-case basis or implement a rule base in which each possibly component is given a weight which might depend on the presence of other arguments. Although the weighting given to each argument is a numerical value, again in practice only certain values might be allowed in order that unjustified precision is not brought into the reasoning.

**Figure 14:**
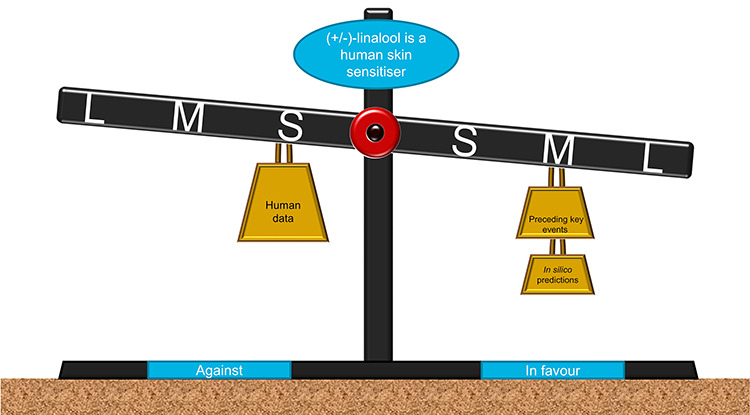
Cartoon representation of a balance of evidence. See accompanying text for description and interpretation of this Figure.

In Fig. 14, a more detailed, though more complex, representation of the arguments is shown, again for the argument leading directly to skin sensitization in human by (+/−)-linalool, the right-most step in Fig. 11. Here the arguments are shown as weights on an arm balance and consist of the direction of the argument (for or against), the intrinsic convincingness of the argument as shown by the size of the weight and the reliability of the evidence about that argument, as shown by the position of the weight on the arm. The lines of reasoning in Fig. 14 can be compared with how they are captured in Figs 11 and 13.

It is worth considering the representation of arguments and how to combine and weight evidence from different (potentially conflicting) skin sensitization assays as shown in Figs 13 and 14 with defined approaches for addressing this problem [59, 60]. There is, currently, no settled view on this, but the different approaches can nevertheless be represented using the approaches shown in Figs 13 and 14.

In representing the differences in convincingness, whilst ensuring there is no unjustified precision, the weights are of three sizes: the most convincing, human data, being the largest and the less convincing, *in silico* prediction, being the smallest. The second dimension of the arguments, the reliability of the evidence, is represented by the position of the weights on the balance; as with convincingness there are only three possible values on the scale large, medium and small. In this case, the human data are considered poorly reliable as it is by nature relatively irreproducible (and indeed, patch studies show about 2% of people do show skin sensitization by oxidation products of linalool). In contrast, the evidence of the preceding KEs is considered more reliable as the assays on which the conclusion is drawn have known levels of repeatability and have been performed several times. Finally, the *in silico* prediction—in this case from Derek Nexus—is considered very repeatable. Figure 14 shows the balance of the arguments by the angle at which the arm balance is deflected: in this case the evidence shows a deflection towards considering linalool to be a human skin sensitizer.

The advantage of considering the convincingness of the argument and the reliability of the evidence for it separately is that they can be recorded and manipulated independently of each other. For instance, the convincingness of an argument may be constant but the reliability of the evidence supporting it may change. The disadvantage is that—as both qualities have a subjectivity to them—it is necessary to define the values that these qualities can take and how they interact with each other, as well as how to calculate an overall conclusion. Furthermore, the definition of these values must be consistently applied throughout the knowledge base, i.e. for both the innate level of belief in the KEs and KERs and the conclusions to be drawn from assay results and *in silico* predictions. Another disadvantage, particularly important in communicating the overall conclusion to a user of software, is that reasoning process is more complex than in the simple conservative approach or a weighting scheme using a single dimension as shown in Fig. 13.

Tables 2 and 3 describe examples of how values for the convincingness of an argument and the consistency of evidence are arrived at and Table 4 contains the positions of all the arguments at all the steps in Fig. 11—though note that only those directly relevant to coming to a conclusion about the level of belief in a particular KE will be used at any one time. We have taken the weights of evidence of the KERs from AOPWiki [8] and using the criteria described in Meek *et al.* [56] to capture the innate KERs.

**Table 2:** Examples of large, medium and small levels of convincingness of arguments

	Convincingness of argument
Large	A direct observation or measurement of the event or AO by *in vivo* assay Measurement of an *in vivo* marker An OECD or similarly approved AOP, KE or KER with strong WoE A conclusion from an approved IATA
Medium	An indirect *In vivo* measure. An *in vitro* measure An OECD or similarly approved AOP, KE or KER with strong WoE
Small	An *in silico* measure

**Table 3:** Examples of large, medium and small levels of consistency of evidence

	Consistency of evidence
Large	Repeated concordant measurements Deterministic (i.e. non-stochastic) model prediction A model prediction for an assay, KE or AO which has high accuracy (as measured by Cooper statistics)
Medium	Reproducible measurements with explainable and interpretable differences Stochastic model prediction with well-defined parameters A model prediction which has good accuracy
Small	Single measurement of unknown reproducibility A model prediction of moderate accuracy

**Table 4:** Knowledge and data about the skin sensitization of (+/−)-linalool expressed in terms of convincingness of the argument and consistency of evidence

		Convincingness of argument		
		Large	Medium	Small
Consistency of evidence	Large	**Assay**: DPRA result **AER**: DPRA predicts for covalent protein binding **KER**: covalent protein binding leads to dendritic cell activation **KER**: covalent protein binding leads to keratinocyte activation **Assay**: KeratinoSens™ result **AER**: KeratinoSens™ predicts for keratinocyte activation **Assay**: U-SENS result **AER**: U-SENS predicts for dendritic cell activation **Assay**: h-CLAT result **AER**: h-CLAT predicts for dendritic cell activation **KER:** dendritic cell activation leads to T-cell activation and proliferation **Assay**: LLNA result **AER**: LLNA predicts for T-cell activation and proliferation **KER:** T-cell activation and proliferation leads to skin sensitization **AER**: Human data predicts for skin sensitization in humans	**KER:** Keratinocyte activation leads to dendritic cell activation	**Prediction:** Derek Nexus prediction
	Medium			**PER:** Derek Nexus prediction reflects skin sensitization in humans
	Small	**Assay**: Human data		

AER is assay event relationship; PER is prediction event relationship.

Once the values that each quality (convincingness of the argument and reliability of the evidence) can take have been defined, it is necessary to define how the two interact, how the argument is transmitted through the network and how independent arguments can be combined. We term the property from the convincingness of the argument and the reliability of the evidence as the Strength of the argument. It is to be noted that ensuring that arguments are indeed independent is quite difficult. In our example of human skin sensitization by linalool, it is possible that the *in silico* prediction is informed by the assay data that have contributed to the conclusions about both the hazard identified from preceding KEs and/or the human data for linalool.

Although it would be possible to convert the values of each quality into numbers and use an operation such as multiplication to combine them in order that an overall contribution from a line of argument be arrived at, this can quickly lead to unjustified levels of precision. It also begs the question as to whether any scale that contains all the possible values for a quantity is linear, with values evenly spaced along it, or irregular, where one value might always outweigh any others (c.f. how ‘Certain’ and ‘Impossible’ in Derek Nexus can never be downgraded by weaker arguments and when put against each other can only be resolved by invoking contradiction rather than equivocacy [61, 62]).

In Table 5, a weighting scheme is suggested which produces a value for a strength of argument from the two separate components. Strength then can take one of five values from the set (Very high, High, Medium, Low and Very low).

**Table 5:** A weighting schema for combining the convincingness of an argument and the reliability of the evidence supporting it

Strength		Convincingness		
		Large	Medium	Small
Reliability of evidence	**Large**	Very high	High	Medium
	**Medium**	High	Medium	Low
	**Small**	Medium	Low	Very Low

Once the strength of an argument is arrived at, it needs to be transmitted through the network. Using the classifications from Meek *et al*. [56] to define the strength of arguments innate to KERs we can then take the lower value of the argument and the connection to the next event to transmit the argument through the network. In our example of skin sensitization of humans by (+/−)-linalool, the Derek Nexus prediction is considered to be of medium strength, but the prediction event relationship for Derek Nexus alert by themselves is considered to be low due to a medium consistency of evidence (the accuracy of a Derek Nexus prediction is actually quite high, but not correct enough to be considered to have high consistency).

Next there is the issue of how independent lines of reasoning combine with each other to produce an overall conclusion. Table 6 shows a proposed method for combining values of strength: small values of strength can combine into higher ones, but only so far: arguments of very low strength do not enhance arguments of medium strength and higher; arguments of low strength also do not enhance arguments of high strength.

**Table 6:** Showing the combination of any two values of argument strength

+(Strength, strength)	Very High	High	Medium	Low	Very Low
**Very High**	VH	VH	VH	VH	VH
**High**	VH	VH	VH	H	H
**Medium**	VH	VH	H	H	M
**Low**	VH	H	H	M	M
**Very Low**	VH	H	M	M	L

When combining arguments, the method is to sort the strengths from lowest to highest and combine them sequentially. For example sequential VL strengths are combined in Equation 1 which shows with parentheses the order in which the pairs of arguments are assessed. Equations (2)–(4) show successive resolving of the innermost pair of arguments: Equation (2) shows two VL arguments resolving to an L argument and Equation (3) shows an L and a VL argument resolving to an M argument. Equation (4) shows that an M argument and a VL argument resolve to an M argument, i.e. there is no increase in the overall strength of the argument by additional VL arguments.

(1) \begin{equation*} \mathrm{VL}+\mathrm{VL}+\mathrm{VL}+\mathrm{VL}=\left(\left(\mathrm{VL}+\mathrm{VL}\right)+\mathrm{VL}\right)+\mathrm{VL} \end{equation*}

 

(2) \begin{equation*} \left(\left(\mathrm{VL}+\mathrm{VL}\right)+\mathrm{VL}\right)+\mathrm{VL}=\left(\mathrm{L}+\mathrm{VL}\right)+\mathrm{VL} \end{equation*}

 

(3) \begin{equation*} \left(\mathrm{L}+\mathrm{VL}\right)+\mathrm{VL}=\mathrm{M}+\mathrm{VL} \end{equation*}

 

(4) \begin{equation*} \mathrm{M}+\mathrm{VL}=\mathrm{M} \end{equation*}

When considering opposing arguments, the prototype software will ‘cancel’ arguments of equal strength but in different directions, combine the outstanding strengths in each direction and then combine them using the difference.

For example in Fig. 14 the method of combining the arguments is shown in Equation (5). Here the −M and +M arguments are cancelled leaving only one +L argument.

Human data, strength = −M

Preceding KEs, strength = +M

*In silico* prediction = +L

(5) \begin{equation*} -\mathrm{M}+\mathrm{M}+\mathrm{L}->\left(-\mathrm{M}+\mathrm{M}\right)+\mathrm{L}=\mathrm{L} \end{equation*}

As can be seen from Table 7, even very strong arguments can be offset by equally strong arguments against and there are no irrefutable arguments. In previous argumentation schemes irrefutable arguments have had a place and combining them can lead to contradiction [62]. Clearly therefore the suggested argumentation scheme is not appropriate where irrefutable arguments may be encountered; irrefutable arguments are rarely encountered in the context of reasoning about toxicity pathways.

**Table 7:** Showing the combination of opposing argument strengths

+(Strength, strength)	Very High	High	Medium	Low	Very Low
−Very High	0	−H	−VH	−VH	−VH
−High	H	0	−M	−M	−H
−Medium	VH	M	0	−VL	−L
−Low	VH	M	VL	0	−VL
−Very Low	VH	H	L	VL	0

Finally, the value of the overall conclusion needs to be put into the context of all possible values of conclusions. In Fig. 14, this is shown by the deflection of the arm balance and the scale of possible values of the overall conclusion can be considered as a torque on the balance, with more powerful conclusions tipping the balance more heavily. From the set of values the argument strength can take, {VH, H, M, L VL, 0, −VL, −L, −M, −H, −VH}, the balance arm can take of 11 positions: five levels of deflection in either direction and perfectly balanced. The overall conclusion for human skin sensitization by linalool, is the second smallest possible value and this can be represented by the small deflection of the scales in Fig. 14.

In this discussion we have presented only straightforward arguments for or against a conclusion. The reality is more complex though and includes such concepts as undercutting arguments (‘gotchas’) where there is good reason to believe that an argument does not apply—for example an assay being used for compounds where it is known to be inaccurate or inappropriate. Although these undermining arguments can be ‘wrapped up’ in a conclusion about a line of reasoning it would be preferable if the user were made aware of them explicitly. In the metaphor of the arm balance, such undercutting arguments could be represented as reducing the convincingness of an argument, but we have not illustrated them in this paper for the sake of simplicity.

## Discussion

In going beyond AOPs to produce reasoned networks for understanding the influences on and likelihood of an AO being observed we have considered three main areas: (i) considering networks rather than single pathways which, though not novel, is a prerequisite of the other aspects of the work; (ii) introducing data and knowledge to the network and (iii) reasoning between the different inputs. The approaches reported in this paper are designed to enable decisions to be made and defended: both low risk (prioritization of resources) and high risk (to defend a decision to a regulatory body when considering the level of risk if exposed to a human). In order to do so, evidence must be curated, organised, and consistently processed in a pre-defined logical and transparent manner so that the user focusses on the data and knowledge and not on the reasoning.

Visualization is important because must allow a holistic and a detailed view depending upon the interest of the user—ideally where it is intuitive and informative, so the focus is on the details and not on how to manipulate it—this becomes increasingly important as the complexity of the network increases.

Going from single pathways to networks causes issues to do with levels of complexity which we have to overcome if the data and knowledge are to be presented to the user in a way i.e. useful rather than cumbersome. Simple scroll and zoom to allow the user to focus in on the area of a network that they are interested in only goes so far: even with an accompanying overall view window that allows the user to keep track of their location within the network, it is likely that the user will get lost in any network than goes beyond the very straightforward. The most noteworthy feature then is the defining and handling of event groups which allows a scientific simplification of the displayed information yet allows the user to drill down where necessary. Event groups—like KEs themselves—have a certain subjectivity to them in where they begin and where they end but well-defined KEGs should make scientific sense.

The use of event groups also allows the better sharing of KEs between networks/pathways. Where similar events are defined in networks leading to different AOs—and therefore will likely be defined by different scientists—whether or not a KE, represented by its name, is the same in two different pathways is a difficult decision to make and a likely source of error and misunderstanding when sharing data and knowledge about KEs. One example might be oestrogen receptor binding, which, notwithstanding the level of detail needed for regulatory acceptance, may, when studied by one group be appropriate to consider at a coarse level, but when studied by a different group needs to be considered at a higher level of refinement/precision (e.g. oestrogen receptor alpha, beta etc.) the use of event groups allows us to do this and to create overlapping event groups which will contain different sub-events as is appropriate for the endpoint under study. To some extent, the use of event groups mirrors the hierarchical and specialization relationships that can be captured with an ontology. We have not developed ontologies for KEs as it is currently beyond the needs of the work we are doing, whilst also finding that public ontologies related to KEs are not yet sufficiently developed for the purposes of arranging and displaying KEs appropriately. We note that, while KEGs may contain ontological relationships, the network or pathway connectedness of the lower level groups is not easily expressed in ontologies where the concept of the ordering of the sequence of the lower level events cannot be captured.

The method of combining weights of evidence from different, ideally independent, lines of reasoning about the likelihood of a KE demonstrates the need for expert opinion on how convincing a line of reasoning might be. This is realised with a weighting scheme that must be well defined in order that it can be applied in different situations. The components of the weighting scheme—which in the case presented are the direction of the argument, the convincingness of the argument and the number of observations of the argument are not the only possible ones, but our experience is that more components to the model does not lead to more convincing conclusions: the user needs to understand the reasoning behind a conclusion whilst being comfortable with the mathematics behind it. The different components of the reasoning model have very restricted values: large, medium and small. This is because a greater degree of precision is not merited in the arguments that are being advanced. By extension, the number of possible values that the reasoning outcome can take is similarly restricted, and again the precision of the value of the reasoning outcome needs to be understood to be small. A comparison can be made with a Bayesian model in which the final reasoned value can be any number in the set of reals. We feel that the arguments advanced in these models do not qualify for that degree of precision—a Bayesian model built with a restricted number set would be needed.

Although in this paper the software support for using AOPs and KENs etc. is demonstrated with a prototype tool, some of the functionality described herein is available through Lhasa Limited’s Kaptis software [63].

## Conclusions

We have shown how we can augment AOP concepts of MIEs, KEs, KER’s and AOs with knowledge about, and data from, assays and (Q)SAR predictions. The knowledge and data allow us to consider which assays might be of particular interest when making an assessment of the toxicity of a chemical, and which assays might be considered to be the most informative to perform next. The construction of prototype software to allow the user to leverage the knowledge and data to hand presents its own challenges in appropriate display and usable interactions by the user.
